# Lack of Correlation between Outcomes of Membrane Repair Assay and Correction of Dystrophic Changes in Experimental Therapeutic Strategy in Dysferlinopathy

**DOI:** 10.1371/journal.pone.0038036

**Published:** 2012-05-29

**Authors:** William Lostal, Marc Bartoli, Carinne Roudaut, Nathalie Bourg, Martin Krahn, Marina Pryadkina, Perrine Borel, Laurence Suel, Joseph A. Roche, Daniel Stockholm, Robert J. Bloch, Nicolas Levy, Rumaisa Bashir, Isabelle Richard

**Affiliations:** 1 Généthon, CNRS UMR8587, Evry, France; 2 Département de Génétique Médicale, Hôpital d’Enfants de la Timone, AP-HM, and Inserm UMR_S 910, Faculté de Médecine Timone, Université de la Méditerranée, Marseille, France; 3 Department of Physiology, University of Maryland, School of Medicine, Baltimore, Maryland, United States of America; 4 School of Biological and Biomedical Sciences, University of Durham, Durham, United Kingdom; Medical College of Georgia, United States of America

## Abstract

Mutations in the dysferlin gene are the cause of Limb-girdle Muscular Dystrophy type 2B and Miyoshi Myopathy. The dysferlin protein has been implicated in sarcolemmal resealing, leading to the idea that the pathophysiology of dysferlin deficiencies is due to a deficit in membrane repair. Here, we show using two different approaches that fullfiling membrane repair as asseyed by laser wounding assay is not sufficient for alleviating the dysferlin deficient pathology. First, we generated a transgenic mouse overexpressing myoferlin to test the hypothesis that myoferlin, which is homologous to dysferlin, can compensate for the absence of dysferlin. The myoferlin overexpressors show no skeletal muscle abnormalities, and crossing them with a dysferlin-deficient model rescues the membrane fusion defect present in dysferlin-deficient mice *in vitro*. However, myoferlin overexpression does not correct muscle histology *in vivo*. Second, we report that AAV-mediated transfer of a minidysferlin, previously shown to correct the membrane repair deficit *in vitro*, also fails to improve muscle histology. Furthermore, neither myoferlin nor the minidysferlin prevented myofiber degeneration following eccentric exercise. Our data suggest that the pathogenicity of dysferlin deficiency is not solely related to impairment in sarcolemmal repair and highlight the care needed in selecting assays to assess potential therapies for dysferlinopathies.

## Introduction

Mutations in dysferlin have been shown to lead to a variety of muscle phenotypes that range from mild to severe, including the prominent Limb-Girdle Muscular Dystrophy type 2B [LGMD2B; OMIM 253601; [1]] and Miyoshi Myopathy (MM, OMIM 254130, [2]]. These muscular dystrophies (MD) are characterized by progressive muscle atrophy and weakness arising in the late teens but differ in the pattern of muscle involvement: proximal in LGMD2B and distal in MM. Electron microscopy analysis of muscle biopsies from LGMD2B or MM patients reveals numerous structural membrane defects such as disruption of the plasma membrane, alterations in the basal lamina and subsarcolemmal accumulation of vesicles [3]. Both naturally occurring and genetically engineered murine models of dysferlin deficiency also show dystrophic changes and loss of membrane integrity [4], [5], [6]. Dysferlin deficiency in these models has been associated with defective membrane resealing of isolated skeletal muscle fibers, a calcium-dependent process involving recruitment of internal vesicles at the site of membrane damage and fusion with the plasma membrane [7]. Changes in the immune system, including increased phagocytic activity in dysferlin-deficient macrophages and enhanced susceptibility to complement attack, have also been proposed to exacerbate the pathophysiology of the disease [8], [9], [10]. However, restoring dysferlin in muscle alone is sufficient to mitigate the dystrophic phenotype [11] suggesting that the primary defect due to the absence of dysferlin lies within the muscle fibers themselves, and not in cells of the immune system.

Dysferlin is a ∼230 kDa membrane-anchored protein in the ferlin family that is composed of a C-terminal transmembrane domain and a very long N-terminal cytoplasmic domain with up to 7 predicted C2 domains (C2A to C2G) that are classically involved in the calcium dependent binding to phospholipids and proteins [12], [13]. Dysferlin is expressed strongly in skeletal muscle, heart, placenta and monocytes [1], [2], [14]. Myoferlin, the ferlin with the greatest sequence homology to dysferlin (56%), is expressed in placenta, heart, lung, endothelial cells but also, at lower levels, in cardiac and skeletal muscles [15]. In these latter tissues, the two ferlin proteins are associated with plasma and T-tubule membranes, with an additional association with nuclear membrane for myoferlin [15], [16], [17], [18]. Although mutations in myoferlin have not been associated to date with a human disease, elimination of myoferlin in mice by homologous recombination leads to a dystrophic phenotype with smaller myofibers and a reduced capacity to regenerate after injury [19]. In addition to previously reported role of dysferlin in membrane repair [6] and of myoferlin in myoblast fusion [19], recent data support a role of these two ferlins in intracellular vesicle trafficking [20].

No treatment is available for dysferlinopathies and gene therapy is complicated by the fact that the dysferlin cDNA is large. However, a strategy based on the capacity of the genomes of different adeno-associated viruses (AAV) to concatemerize after transduction restored the ability of the sarcolemma to repair in *in vitro* assays and improved muscle function *in vivo* [21]. Identification of mutations in dysferlin associated with mild phenotypes led to the suggestion that exon skipping [22], [23] or use of a small version of dysferlin that would have retained most of the protein’s activity – “a minidysferlin” [24] - hold promise for the development of new therapeutic strategies.

In this report, we used transgenic mice to learn if over-expression of myoferlin can compensate for dysferlin deficiency. We observed that myoferlin overexpression can compensate for the membrane fusion defect seen in laser wounding assays of dysferlin-null muscle fibers *in vitro* but it did not improve muscle histology *in vivo*. Similarly, a form of minidysferlin, cloned from a patient with a mild clinical presentation, expressed in muscle *via* an AAV vector, although previously shown to be efficient in rescuing sarcolemmal repair *in vitro*, did not prevent the development of a dystrophic phenotype in mice. Furthermore, neither of the two therapeutic approaches prevented irreversible damage to muscle following eccentric exercise *in vivo*. Consequently, the *in vitro* impairment of sarcolemmal resealing associated with dysferlin deficiency is likely a by-stander effect that is not directly linked to the pathogenicity.

## Results

### Design and Characterization of the Myoferlin Transgenic Mice

**Figure 1:**
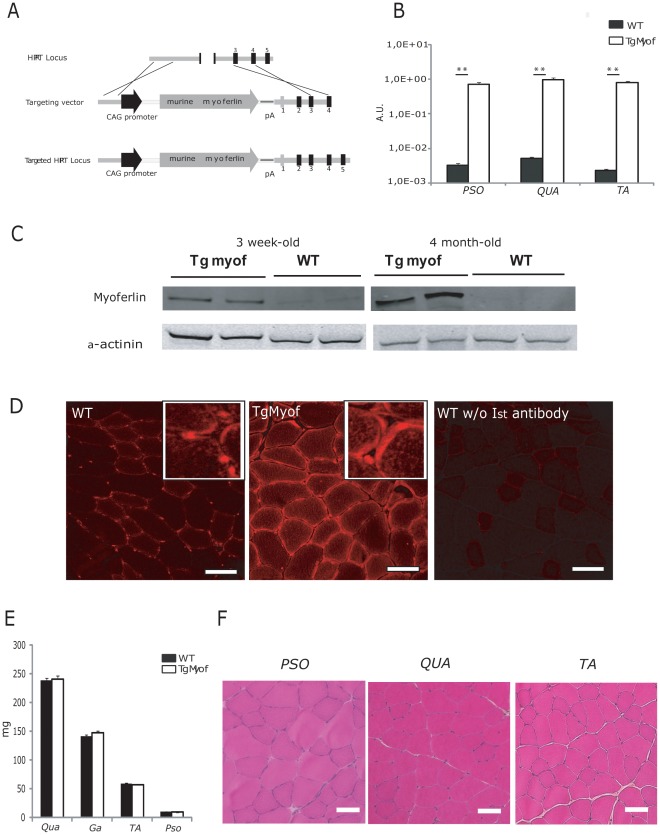
Characterization of the Myoferlin transgenic mice. A /Schematic representation of the Myof construct and Hprt targeted allele. **B**/Quantitative RT-PCR for myoferlin expression in muscles of WT and TgMyof revealed a 200-fold greater expression in the transgenic. The data are presented relative to a titration with the original myoferlin expression plasmid. (** *p<*0.01 between WT and TgMyof). **C**/Western blot for the myoferlin protein in WT and TgMyof showing the overexpression in the transgenic animals (results for 2 animals at 3 weeks and 4 months of age are shown). A-actinin staining was used to confirm that similar amounts of total protein extracted from muscles were loaded for all samples. **D**/Immunofluorescence of myoferlin in sections of WT (left panel) and TgMyof (middle panel) muscles. A control in which the primary antibody was omitted is shown (right panel). Scale bar=50 µm. **E**/Weight of WT and TgMyof muscles at 9 months of age. The values of TgMyof muscles are not statistically significant compared with WT mice (*p*>0.05). **F**/Histological analysis of 9-month-old male TgMyof showed no abnormality. Scale Bar=50 µm.

To test the hypothesis that myoferlin, the closest member of the ferlin family to dysferlin, could compensate for dysferlin deficiency, we developed a transgenic mouse carrying the murine myoferlin cDNA under the transcriptional control of the chicken β-actin (CAG) promoter at the hypoxanthine-guanine phosphoribosyl transferase (*hprt*) locus (Figure 1A).

The resulting model, which we refer to as TgMyof, produces litters of normal size, sex ratio and longevity. We analyzed myoferlin mRNA expression by quantitative RT-PCR (qRT-PCR) in 6-month-old male animals to determine the extent of over-expression of myoferlin (Figure 1B). In the three muscles examined [*Psoas* (*Pso*), *Quadriceps* (*Qua*) and *Tibialis Anterior* (*TA*)], the mRNA encoding myoferlin was overexpressed in the transgenic line more than 200-fold compared to C57BL/6. When quantified on serial dilutions of the myoferlin plasmids, the mean copies number in the WT and TgMyof was defined as 4×10^7^ (±3.9×10^6^) and 10×10^10^ (±6.7×10^8^) copies per µg RNA. We then analyzed protein expression by western blotting with a myoferlin antibody using TA muscles from mice at two different ages (3 weeks and 4 months). We observed that myoferlin is more expressed in young WT mice and is barely visible in older mice (Figure 1C). Quantification indicated that myoferlin protein levels were 4 and 100 times higher in muscles of the transgenic mouse at 3 weeks and 4 months of age, respectively, compared to WT (*p*<0.05). Immunostaining experiments performed on *PSO* sections showed that myoferlin was concentrated in the sarcolemmal region of the fibers (Figure 1D, middle panel). This localization was not different from what was observed in the wild-type animals (Figure 1D, left panel). In subcellular fractionation experiments, myoferlin was found in the same sub-cellular compartments (cytosol, membrane and nuclear fractions) in TgMyof and WT muscles (Figure S1A). No significant difference in skeletal muscle weight was observed between TgMyof and C57BL/6 (Figure 1E). No histopathological abnormal features, including centrally nucleated fibers (CNF) could be observed in the skeletal muscle of these mice in Hematoxylin-Phloxine-Saffron (HPS) stained sections from 9-month-old animals (Figure 1F). The contractile function of TgMyof muscles was compared with WT muscles at 2 months of age using a tetanic stimulation for 300 ms (n=4). The maximal tetanic torques generated by the anterior compartment of the leg of TgMyof and WT (2.8±0.6 and 2.3±0.5 N.mm, respectively) or the ratios of torque to the weight of the muscles (59±10 and 43±10 N.mm/g, respectively) were not statistically different (Figure S1B).

### Myoferlin Overexpression Restores Sarcolemmal Repair in Dysferlin-deficient Muscle

**Figure 2:**
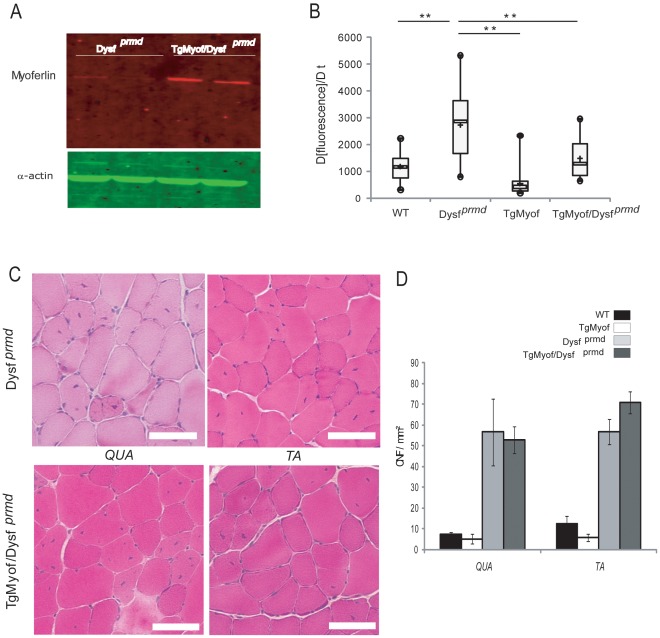
Myoferlin overexpression improves sarcolemmal repair after laser wounding but do not improve muscle histology. A /Western blot for the myoferlin protein in *Dysf ^prmd^* and TgMyof/*Dysf ^prmd^ FDB* muscles shows the overexpression of myoferlin in TgMyof/*Dysf ^prmd^* (results for 2 animals are shown for each muscle and condition). A-Actin was used as loading control. **B**/Box plots represent the rate of change of fluorescence (Δ[fluorescence]/Δt) in the fibers of WT (n=29), *Dysf ^prmd^* (n=14), TgMyof (n=31) and TgMyof/*Dysf ^prmd^* (n=17). Boxes extend from the 25th to the 75th percentile values. Minimum and maximum values are indicated by the dots at the end of the vertical lines. Horizontal bold bars indicate the median value. **p<0.01 compared to dysferlin-deficient mice. Results indicate that overexpression of myoferlin restores membrane repair in the absence of dysferlin. **C**/Histological analysis of *Qua* and *TA* of 6-month-old *Dysf ^prmd^* and TgMyof/*Dysf ^prmd^*. Necrotic fibers, CNF, cellular infiltrations and fiber size variations are seen in *Dysf ^prmd^* and TgMyof/*Dysf ^prmd^* muscle. Scale bar=50 µm. **D**/CNF were counted in HPS-stained transverse cryosections from WT, TgMyof, *Dysf ^prmd^* and TgMyof/*Dysf ^prmd^* 6-month-old mice. The number of CNF in TgMyof/*Dysf ^prmd^* are not significantly different when compared with *Dysf ^prmd^* mice.

To determine whether increased myoferlin levels could ameliorate the phenotype resulting from dysferlin deficiency, the transgenic mice were crossed with dysferlin-deficient mice [B6.A/J-*Dysf ^prmd^*] to generate TgMyof/*Dysf ^prmd^*. We verified by western blot analysis that myoferlin was still overexpressed (Figure 2A). We analyzed the sarcolemmal repair capacity in isolated *Flexor Digitorum Brevis* (*FDB*) fibers of 1-month-old animals by performing laser-wounding in the presence of FM1-43 dye, as previously described [6], [25]. In this assay, isolated muscle fibers from WT mice successfully repair membrane lesion in the presence of calcium, as seen by the rapid halt of dye entry, whereas *Dysf ^prmd^* fibers do not (Figure 2B). Remarkably, fibers from TgMyof as well as from TgMyof/*Dysf ^prmd^* responded to laser wounding like wild-type mice, by showing rapid stabilization of intracellular fluorescence due to FM1-43 (Figure 2B). This result indicates that expression of myoferlin in the absence of dysferlin was able to promote sarcolemmal repair after laser wounding.

### Improvement of Membrane Repair Capacity does not Result in Reduction of Dystrophic Changes in Dysferlin-deficient Muscle

To analyze whether the restoration of membrane repair capacity was associated with improvement of muscle health, we investigated the impact of myoferlin overexpression at the histological level in dysferlin-deficient muscles. *Qua* and *TA* muscles from TgMyof and TgMyof/*Dysf ^prmd^* were sampled in 6-month-old animals. Examination of HPS-stained sections showed that the histology of the muscle was not improved with the overexpression of myoferlin, despite their improved response to laser wounding, since they showed a high level of dystrophic features (Figure 2C). In particular, CNF were at similar levels to those of age-matched *Dysf ^prmd^* mice (Figure2D). These results suggest that overexpression of myoferlin is not sufficient to correct the dystrophic phenotype associated with dysferlin deficiency.

**Figure 3:**
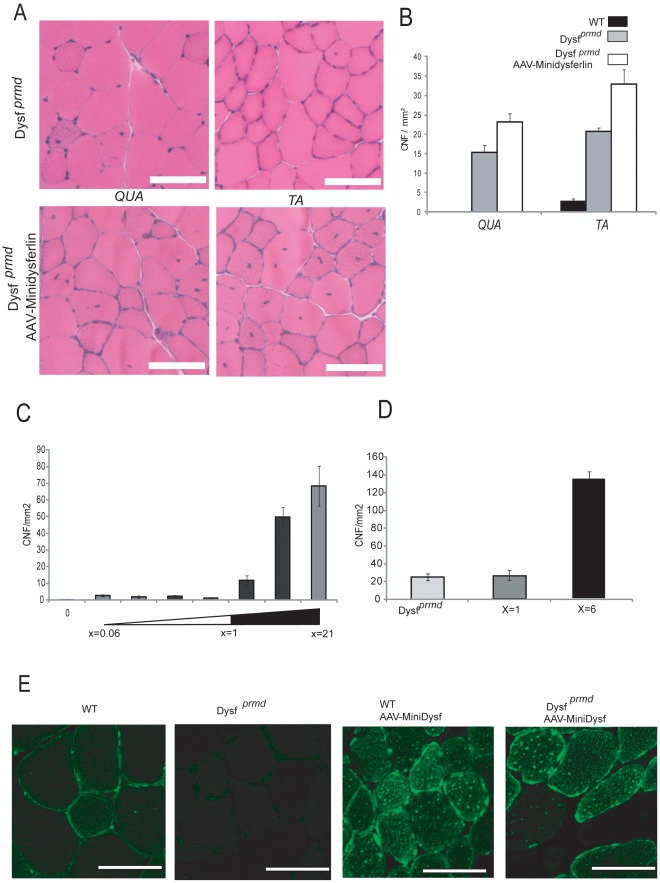
Minidysferlin do not improve muscle histology. A /Histological analysis of Q*ua* and *TA* of injected and non-injected 2-3-month-old *Dysf ^prmd^* mice by HPS staining. Necrotic fibers, CNF, cellular infiltrations and fiber size variations are seen in both injected and non-injected *Dysf ^prmd^* mice. Scale Bar=100 µm. **B**/CNF were counted in HPS-stained transverse cryosections from injected and non-injected *Dysf ^prmd^* mice and normalized to the surface area of the muscle. No reduction in number of CNF in *Dysf ^prmd^* mice after AAV-mediated transfer of minidysferlin was observed (*p*>0.05). **C**/Level of CNF in *TA* muscles of WT animals injected with different doses of two different preparations (prep.1 and prep.2) of AAV-minidysferlin. The level of minidysferlin expression was quantified by qRT-PCR and compared to the level of endogeneous dysferlin in a normal human muscle. The ratio is indicated below the graph. The results are ordered according to the dysferlin expression level that was obtained. **D**/Quantification of CNF in muscles of *Dysf ^prmd^* mice that expressed an equivalent level of minidysferlin than dysferlin in normal muscle and 6 times this dose. No decrease in the number of CNF was seen with the dose giving a level of minidysferlin equivalent to the dysferlin expression (x=1) whereas an important increase in seen with the dose leading to a 6-fold increase of expression (x=6). It should be noted that this fold correspond to a mean expression and that each individual fiber can express more or less than this means. **E**/Immunofluorescence of dysferlin in sections of muscles in the *Dysf ^prmd^* and WT mice. The non injected muscles are on the left of the figure and the muscles injected with AAV-minidysferlin on the right. Spots of intense fluorescence were seen throughout the cytoplasm in injected conditions, suggesting accumulation/aggregation of the minidysferlin protein. Scale bar=50 µm.

We next examined if a naturally occurring deletion variant of dysferlin (Figure S2A) could restore dysferlin-null muscles to health. This minidysferlin was originally identified in a patient with a mild form of dysferlinopathy with little dystrophic features, in particular with a very low level of CNF and shown to restore the membrane repair capacity of dysferlin-null muscle, following laser wounding [24]. Four-week-old *Dysf ^prmd^* male mice were injected in the left *TA* with rAAV2/1 vectors [1.2×10^9^ viral genome (vg)] carrying the minidysferlin under the control of the C5-12 promoter. The TA muscles were sampled 7 weeks after injection. RT-qPCR performed with primers at the end of the dysferlin cDNA showed that 20-fold more minidysferlin is expressed compared to amount of full-length dysferlin in a normal human skeletal muscle (data not shown). Immunoblot analyses were also performed on extracts of these muscles and confirmed the upregulation of the minidysferlin compared to endogenous dysferlin (Figure S2B). Similarly to what we found with overexpression of myoferlin, the histology of the transduced muscles was not improved since HPS-stained sections showed a high level of dystrophic features, including many fibers with centrally located nuclei (Figures 3A and B). A possible toxic effect of minidysferlin overexpression can even be noted since the level of centronucleation is significantly higher in the injected *Dysf ^prmd^* than in the noninjected muscles (*p*<0.05).

To ascertain this possible toxic effect, we injected different doses (from 9.3×10^7^ to 1.6×10^10^ vg) of AAV-minidysferlin in TA of WT animals and sampled the muscles 4 weeks later. We used two different viral preparations to circumvent any possible effect due to the quality of the preparation. The level of expression of minidysferlin was quantified by qRT-PCR and ranged from 0.06 to 21 fold compared to dysferlin expression in a healthy human muscle control. Morphometrical analysis of HPS-stained muscle sections showed that the muscles injected with the lowest doses were completely normal and that the muscles injected with doses that lead to an expression of minidysferlin higher than a corresponding level of dysferlin in normal muscle exhibit a number of CNF that increase with the dose (Figure 3C). We performed a similar experiment with *Dysf ^prmd^* mice and found that an expression of minidysferlin equivalent to the level of dysferlin in normal muscle did not lead to a modification of the number of CNF in *Dysf ^prmd^* muscle (Figure 3D). This observation suggests that even a non toxic dose of minidysferlin is not able to reverse the dysferlin related pathology. In contrast, an excess of minidysferlin as low as 6-fold led to an important increase in CNF (Figure 3D). Immunofluorescence detection of the protein was performed in sections of WT and *Dysf ^prmd^* muscles non injected and injected with high doses of AAV-minidysferlin. Spots of intense fluorescence were seen throughout the cytoplasm in injected conditions, suggesting accumulation/aggregation of the minidysferlin protein (Figure 3E).

### AAV-mediated Transfer of Minidysferlin and Myoferlin Overexpression do not Prevent Myofiber Degeneration Following Eccentric Contractions in Dysferlin-deficient Muscle

**Figure 4:**
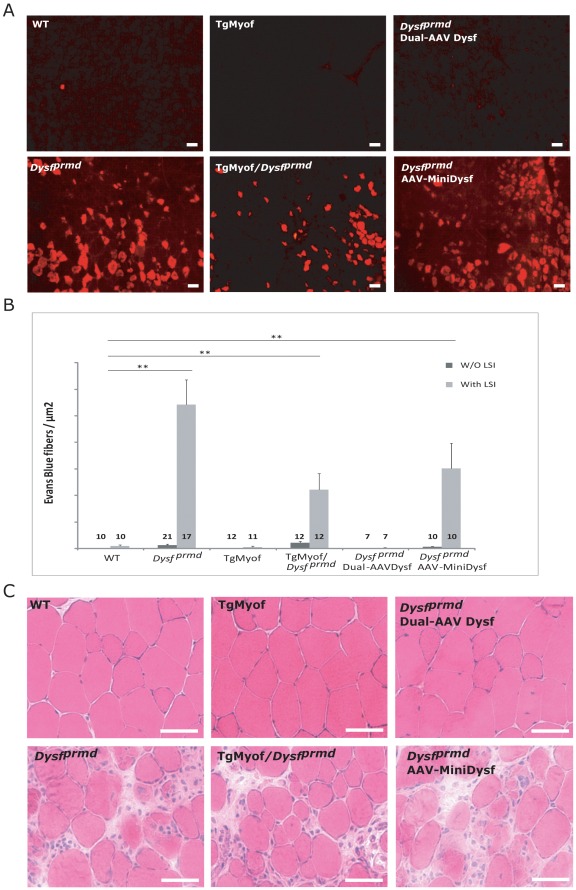
Myoferlin overexpression and Minidysferlin do not prevent myofiber degeneration following eccentric contractions in dysferlin-deficient muscle. **A**/Ten-week-old mice were subjected to LSI and their *TA* muscles were harvested 3 days after injury. EBD was injected 24 hours before tissue collection. Cryosections of *TA* muscles were studied under a fluorescent microscope to visualize Evans Blue dye. A high number of EBD positive fibers were observed in *Dysf ^prmd^*, TgMyof/*Dysf ^prmd^* and rAAV2/1-Minidysferlin-injected *Dysf ^prmd^*. The number of analyzed samples is indicated in the graph. Scale Bar=50 µm. **B**/The number of positive fibers were counted on the whole section and reported to the total area of the section. Numbers of positive fibers are not statistically different in TgMyof/*Dysf ^prmd^* and rAAV2/1-Minidysferlin-injected *Dysf ^prmd^* compared to *Dysf ^prmd^*. **C**/HPS-stained muscle sections of the different conditions.

We studied muscles from animals subjected to our experimental therapeutic strategies for their ability to recover from eccentric exercise using the Large Strain Injury (LSI) model [11], [26], [27]. LSI produces an injury from which normal muscle recovers via local repair without myogenesis. However, the recovery of dysferlin-null muscle is characterized by widespread necrosis and inflammation, which is subsequently followed by myogenesis [26], [27]. In particular, dysferlin-deficient muscle recovers after LSI more slowly than WT and shows much higher level of necrosis 3 days after injury [26]. Four-week-old WT and *Dysf ^prmd^* male mice were injected in the left *TA* with 1.2×10^9^ vg of rAAV2/1-minidysferlin. As a positive control for recovery, a group of *Dysf ^prmd^* mice were injected in the left *TA* with the dual AAV vectors encoding full-length dysferlin (4×10^9^ vg each vector) that promote sarcolemmal repair and correction of muscle pathology [21]. Six weeks after injection, these two groups of mice, together with WT, TgMyof and TgMyof/*Dysf ^prmd^* mice, were subjected to the LSI protocol and allowed to recover for 3 days. Evans Blue Dye (EBD) was injected intraperitoneally one day before sacrifice to follow the post injury necrosis that was previously observed to occur in dysferlin-deficient muscles 3 days after LSI [26]. Left and right (uninjected) *TA* muscles were sampled and effective transfer of rAAV2/1-minidysferlin was validated by western blot (data not shown). Examination of cryosections by fluorescence microscopy revealed no EBD uptake in WT, full length dysferlin or TgMyof muscles (Figures 4A and B). In contrast, minidysferlin-injected and TgMyof/*Dysf ^prmd^* myofibers demonstrated pronounced EBD uptake at levels which were statistically indistinguishable from the *Dysf ^prmd^* mice (*p*>0.05) although the presence of high level of myoferlin seems to have a slight positive effect. Accordingly, the fiber necrosis that is present in dysferlin-deficient muscle 3 days after an *in vivo* injury does not appear to be corrected by over-expression of myoferlin or minidysferlin–yet both facilitate recovery of myofibers subjected to laser wounding *in vitro*.

## Discussion

We investigated the potency of two different strategies for reversing the myopathology of dysferlin deficiency: overexpression of myoferlin and AAV-mediated transfer of a minidysferlin. We used a laser wounding assay of myofibers in tissue culture to assess the efficacy of these treatments in promoting sarcolemmal repair, histology to examine the dystrophic phenotype *in vivo*, and large strain injury to assess the susceptibility of skeletal muscles to damage from eccentric contractions. The results show that the restoration of sarcolemmal repair afforded by each of these therapeutic strategies is not associated with improvement of the histology or full protection against myofiber degeneration observed 3 days after injury from eccentric contractions. These data indicate that dysferlin’s role in skeletal muscle *in vivo* is not effectively assayed by laser wounding of myofibers *in vitro*, and that the evaluation of therapeutic strategies based on that *in vitro* assay should be re-examined.

The skeletal muscles of TgMyof mice overexpress 30-fold more myoferlin than in WT, but are indistinguishable from WT muscles in weight and morphology. Centrally located nuclei, tissue fibrosis and necrosis, typical features of dystrophy, were not observed, even in 9-month-old animals. Interestingly, several lines overexpressing different levels of dysferlin, generated previously [11], [28], can show myopathology, depending on protein dosage. One line with three-fold overexpression was phenotypically indistinguishable from WT with no obvious tissue pathology or no change in weight [11]. In another study, in which 3 lines of mice overexpressing low (2-fold), medium (36-fold) and high (176-fold) levels of dysferlin were examined [28], the low level was well tolerated, but medium and high levels were not. The mice expressing these elevated levels of dysferlin were kyphotic with an irregular gait and a non-necrotic progressive muscular dystrophy characterized by marked atrophy and weakness. From molecular analysis of several proteins, the authors proposed that dysferlin overexpression may alter the stoichiometric assembly of the membrane repair complex or lead to deregulated intrasarcoplasmic Ca2+ homeostasis through generation of the Unfolded Protein Response (UPR) which overwhelms the Endoplasmic Reticulum Associated Degradation (ERAD) machinery [28].

The level of myoferlin overexpression in our transgenic mouse was similar to the medium level of expression of dysferlin of Glover *et al.* [28]. However, this level of overexpression does not lead to morphological abnormality in the case of myoferlin, suggesting that, unlike dysferlin, significant levels of overexpression of myoferlin in wild-type animals are not cytotoxic. One possibility is that expressing myoferlin as a murine protein in mice, as we did, would be less toxic than expressing a human version of dysferlin, as by Glover *et al,* that, in a murine context, would have been more difficult to fold, generating an ER stress response. Of course, even higher levels of myoferlin expression than we have been able to achieve may be cytotoxic. In contrast to overexpression of myoferlin but in accordance with the dysferlin overexpression studies, overexpression of minidysferlin seems also to have a toxic effect starting with level of expression 6 times higher than the level of dysferlin in normal muscle. Since a non toxic dose of minidysferlin was not able to reduce the level of CNF in *Dysf ^prmd^* mice, it can be suggested than no compensation of the dysferlin pathology was brought with the minidysferlin. However, the patient from whom the minidysferlin was derivated shows a moderately severe clinical presentation with a mild dystrophic aspect on the biopsy. Therefore, it seems that a low level of this protein prevents most of the pathological aspects if present from the beginning, This fact also underlines that dysferlin-related membrane fusion events may play an important role during muscle development [18], while dysferlin function in mature muscle would implicate additional pathways.

Our data indicates that myoferlin is competent in repairing membrane tears, even if only partially present at the sarcolemma. Interestingly, this competency may participate in the fact the dysferlin deficient myoblasts exhibit membrane repair as reported [29].since myoferlin is highly expressed in myoblast [19]. Nevertheless, the inability of myoferlin to compensate for dysferlin deficiency despite their similar structure and presumptive fusogenic functions indicates that dysferlin performs functions vital to muscle homeostasis that myoferlin cannot perform. Indeed, the need for two highly homologous proteins in the same tissue argues in favor of unique function with spacio-temporal specify for each of these proteins. Their expression profiles together with experimental data suggest a role for myoferlin in myoblast fusion and for dysferlin in postfusion myotubes and myofibers while both seem implicated in receptor recycling [15], [17], [19], [30], [31], [32], [33]. Further study is needed to ascertain the crucial functions that dysferlin performs that are relevant to the onset and progression of dysferlinopathies.

The differences in function between dysferlin and myoferlin may be due to differences in the proteins with which they interact, preventing a function to be fulfilled or else trapping the proteins in different compartment. Similarly, the inability of minidysferlin to compensate for dysferlin deficiency may also be due to its inability to interact with the same proteins or regulate the same processes as the full-length protein. Several binding partners of dysferlin have been reported to date [34], [35], [36], [37], [38], [39], [40]. However, for all but AHNAK, the relative corresponding interaction with myoferlin is not known. Among dysferlin’s partners, AHNAK and tubulin are known to interact with the N-terminus part of dysferlin. Therefore, their binding sites are likely to be missing in the minidysferlin. These proteins would be the first to be further looked at to define whether the loss of the corresponding interaction would participate in the pathogenesis of dysferlinopathies. On this basis, we are currently developing mididysferlins by adding additional domains to the minidysferlin to promote protein interactions, while maintaining a final size of the corresponding cDNA in the packaging range of rAAV vectors for efficient muscle gene transfer.

Our results indicate that restoration of sarcolemmal repair after laser wounding *in vitro* does not correlate with a decrease in dystrophic changes seen in dysferlin-deficient mice. Therefore, the laser wounding assay does not seem sufficient for determining the functionality of dysferlin in muscle, as it does not report the actual dystrophic state of muscle accurately. This raises questions about the validity of the assay, and whether it is appropriate for studying dysferlin’s function in muscle fibers *in vitro*. It also suggests the need for a different *in vitro* assay that produces results consistent with *in vivo* criteria of human pathology. A deeper understanding of the function of dysferlin with respect to vesicle trafficking or inflammation, for example, may be required for this purpose.

In conclusion, our data demonstrate that myoferlin and a minidysferlin can compensate for dysferlin-deficiency in an *in vitro* assay of sarcolemmal repair but not for the defects in muscle *in vivo* that lead to muscular dystrophy associated with the absence of dysferlin. These observations suggest that, either correction of sarcolemmal repair is not sufficient to prevent pathology or that the laser wounding method, which we and others have used, does not accurately assess the biological activity of dysferlin. Our results highlight the care needed in selecting assays to assess potential therapies for dysferlinopathies.

## Materials and Methods

### *In vivo* Experiments

All procedures on animals were performed in accordance with the directive of 24 November 1986 (86/609/EEC) of the Council of the European Communities and were approved by Genethon’s ethics committee under the number CE11_013 and CE11_014. C57BL/6 mice were purchased from Charles River Laboratories (Les Oncins, France). [Tg(myof)1Iri] mice also called TgMyof were generated by Speedy Mouse® Technology (Nucleis, France) to produce a single-copy transgene insertion at the hprt locus. The coding sequence of murine myoferlin was cloned into the polylinker of the Gateway® pENTR-1A vector (Invitrogen) previously modified to include the ubiquitous chicken β-actin (CAG) hybrid promoter. The transgene was then transferred by Gateway technology into a destination vector pDEST-HPRT (Nucleis) carrying two homologous arms of the *hprt* gene. The vector was linearized using PvuI and electroporated into *hprt* deficient BPES-embryonic stem (ES) cells by standard methods. The targeting construct contained the missing sequences in BPES ES cells and two regions of homology of the *hprt* gene that allowed insertion of the transgene at this locus (Figure 1). The targeted ES clones were efficiently selected, due to the restoration of the ability to grow in Hypoxanthine-Aminopterin-Thymidine (HAT) medium as a result of correct homologous recombination.

Genotyping of HAT-resistant ES clones was performed by PCR analysis of genomic DNA with the following primers: GenetBS2.s 5′-TGCAAGACCATGCGCTTCAT-3′, HPRT.as 5′- GATAGGTCAGGTAAGCAAGCAAC -3′ and HPRTm-int1a 5′- CCCCTTCCCTTTTCTCCCTC -3′ and HPRTm-int1as 5′- CTAAGGCAGGAGGATTCCAG -3′. Using these primers, the wild-type locus gives a band of 300 bp and the mutant form, a band of 1900 bp. Samples were amplified for 30 cycles, each consisting of denaturation at 94°C for 40 s, annealing at 60°C for 40 s, and elongation at 72°C for 40 s. Targeted ES cells were injected into C57BL/6-derived blastocysts that were then transplanted into the uteri of recipient females. Resulting chimeric males were bred with C57BL/6 females, and the N1 agouti female offspring were backcrossed with C57BL/6 males. The resulting heterozygous mice were crossed together; homozygous females resulting from this cross were crossed with hemizygous males to generate the line.

TgMyof/*Dysf ^prmd^* were generated through successive backcrossing of TgMyof males with *Dysf ^prmd^* females. Dysferlin genotyping was performed on tail DNA as described [5]. Hemizygous TgMyof females were crossed with *Dysf ^prmd^* males [25]. Genotyping of the progeny was performed to identify the mice that were homozygous for the *Dysf ^prmd^* mutation while carrying myoferlin transgene.

C57Bl/6 mice were purchased from Charles River Laboratories (Les Oncins, France). *Dysf ^prmd^* mice backcrossed onto C57BL/6 background through N4 were used in this study. The genetic background of TgMyof, TgMyof/*Dysf ^prmd^* and *Dysf ^prmd^* animals were 87.5%, 90.6% and 93.75% of the C57BL/6 background. Mice were injected into the left *TA* with different doses of two different AAV-minidysferlin vectors (9.3×10^7^, 2.7×10^8^, 8.2×10^9^ or 1.6×10^10^ vg for the first preparation and 1.4×10^8^, 4.2×10^8^ or 1.3×10^9^ vg for the second preparation) in 30 µl. The construction and production of the AAV vector was as previously described [24]. Seven weeks after the injection, mice were sacrificed and muscles were removed and quickly frozen in liquid nitrogen-cooled isopentane.

### Messenger RNA Quantification

Total RNA was extracted from muscles lysates by the Trizol method (Invitrogen). Residual DNA was removed from the samples using the Free DNA kit (Ambion). One µg of RNA was reverse-transcribed using random hexamers and the Verso cDNA kit (Abgene). Quantitative RT-PCR (RT-qPCR) analyses were performed using the primer pairs and Taqman probe used for myoferlin as follows. Myof.F: AGGCCCGTGGTGAAAGTT, Myof.R: GGATTGTTCCCTCTCTTGATTCTC and probe Myof.P: CATCTGTGGTCAGACGCACCGCA and for dysferlin as follows. hDysf.F: GGCCTGGACCTCCCTTCA, hDysf.R: TCAGGTCATCCAGGATCACATC and probe hDysf.P: TCACCCCACGGAGAGCCAGAAGG. Total RNA from an adult skeletal muscle served as quantification control (Stratagene 540029-41). The ubiquitous acidic ribosomal phosphoprotein (P0) was used to normalize the data across samples. The global level of overexpression was calculated relative to the quantification on serial dilutions (1×10^4^ to 1×10^10^) of the myoferlin plasmid and reported to one µg of RNA.

### Western Blotting

Muscles were homogenized using FastPrep automated homogenizer (MP Biomedicals, Santa Ana, CA, USA) in lysis buffer (20 mM pH 7.5 Tris, 150 mM NaCl, 2 mM EGTA, 0.1% 100X Triton; 25 µl per mg of tissue) supplemented with Complete Mini Protease Inhibitor Cocktail (Roche) and 2 µM E64 (Sigma). Samples were mixed with loading buffer [NuPage LDS (Invitrogen), DTT 3 M (Sigma)], denatured for 10 minutes at 70°C and briefly centrifuged. Proteins were separated by 3–10% polyacrylamide gradient NuPAGE gels (Invitrogen). After blotting, efficacy of the transfer was verified by Ponceau Red staining (0.2% Ponceau Red/1% acetic acid). Membranes were probed with antibodies against myoferlin (SC51368 polyclonal antibody, Santa Cruz, diluted 1/200), dysferlin (NCL-Hamlet, Novocastra, dilution 1/500), α-actin (A-2066, polyclonal antibody, Sigma, diluted 1/400) or α-actinin (SC15335, polyclonal antibody, Santa Cruz, diluted 1/200) at room temperature for 2–3 hours. Finally, membranes were incubated with IRDye® for detection by the Odyssey infrared-scanner (LI-COR Biosciences, Lincoln, Nebraska, USA). Bound antibodies were detected at excitation wavelengths of 600 or 800 nm. Relative protein levels were quantified with the Odyssey 2.1 software (LI-COR Biosciences).

For subcellular fractionation, after homogenization using FastPrep in lysis buffer supplemented with Complete Mini Protease Inhibitor Cocktail (Roche), proteins were extracted with the ProteoExtract® Subcellular Proteome Extraction Kit (S-PEK, Calbiochem, Germany). Western blots were performed as described above.

### Histology and Immunohistochemistry

Cryosections (8 or 10 µm thickness) were prepared from frozen muscles. Transverse sections were processed for Hematoxylin-Phloxine-Saffron (HPS) staining. For HPS stained sections and centro-nucleated fibers (CNF), digital images were captured using a CCD camera (Sony) and processed using the Histolab software (Microvision, Evry, France).

For immunodetection of myoferlin, sections were dried at room temperature for 10 min. Sections were then incubated in blocking solution (3% BSA in PBS) for 45 minutes. Then, they were incubated over night at 4°C with the monoclonal antibodies against myoferlin (SC-20, SantaCruz, diluted 1/50). After three washes in PBS for 5 min each, sections were incubated with secondary antibodies [donkey anti-goat antibody conjugated with Alexa-594 dye (Invitrogen; dilution 1/1000)]. After three washes in PBS, the sections were mounted with Fluoromount G (SouthernBiotech, Birmingham, USA) and examined under a Leica confocal microscope at 594 nm.

For immunodetection of dysferlin, sections were boiled for 10**minutes in PBS for antigen retrieval, and then cooled for 10**minutes in PBS at room temperature. Sections were incubated in Mouse-On-Mouse blocking reagent (Vector; MBK-2213). Then, they were incubated over night at 4°C with the monoclonal antibody dysferlin (Hamlet, diluted 1/20). After three washes PBS for 5 min each, sections were incubated with biotinylated antibody [F(ab)′2 Goat anti-IgG1Mouse Biot (Southern Biotechnology; diluted 1/200) for 60 min. After three washes in PBS, sections were incubated with streptavidin antibody conjugated with Alexa-488 dye (Invitrogen; S11223; diluted 1/1000) for 20 min. After three washes in PBS, the sections were mounted with Fluoromount G (SouthernBiotech, Birmingham, USA) and examined under a Leica confocal microscope at 488 nm.

Fibers labeled with Evans Blue dye were revealed by fluorescence excitation at 633 nm on a Leica confocal fluorescent microscope. Images from the whole sections were taken using a motorized stage at an original 40X magnification and ratio of the area corresponding to the Evans Blue positive cells versus the whole area of the section was measured using Image J in the red channel.

### Membrane Repair Assay

Membrane repair assays on isolated muscle were performed as previously described [25].

### Injury Model and Assessment of Contractile Function

Assessment of muscle function was performed by measuring the contractile torques during a tetanus induced by a 300 ms pulse trains using a rig previously described [41]. The LSI experiments were performed as described [26]. Briefly, we used 15 lengthening contractions to yield approximately 40% loss of contractile torque in the ankle dorsiflexor group of muscles. For each lengthening contraction, muscles were tetanically stimulated for 300 ms; 150 ms after onset of stimulation, the ankle was plantarflexed from 90 to 175° at an angular velocity of 1200°/s. A 2-min rest between successive lengthening contractions minimized the possible effect of fatigue. Two days after the LSI, the mice were injected intraperitoneally with Evans blue dye (1 mg/g of body weight). The following day, mice were sacrificed and the TA muscles were removed and quickly frozen in liquid nitrogen-cooled isopentane.

### Statistics Analysis

Data are presented as means ± s.e.m. Individual means were compared using the Mann-Whitney non-parametric test. Differences were considered to be statistically significant if *p*<0.05.

## Supporting Information

Figure S1 **Characterization of TgMyof. A/Assessment of myoferlin levels in subcellular fractionations.** The distribution of myoferlin in *TA* muscles was examined by Western analysis of subcellular fractions prepared using SPEK muscle extract from WT and TgMyof mice. Equal volumes of each fraction (nucleus, cytosol, membrane, cytoskeleton) were analyzed and show a similar distribution between fractions despite the overexpression (e.g. presence in the cytosol, nucleus and membrane fraction). **B/Contractile torque of TgMyof and WT.** The anterior compartment of the hindlimb of TgMyof and WT animals was tetanically stimulated for 300 ms and the contractile torques were measured. The differences were not statistically significant.
(TIF)

Figure S2 **AAV vector for minidysferlin. A/Upper scheme:** Scheme of the protein domain organization of the dysferlin protein with its 7 C2 (from C2A to C2G), Fer, Dysf and transmembrane domains. **Middle scheme:** Scheme of the minidysferlin used in this study. **Lower scheme:** Scheme of the rAAV construct for minidysferlin. **B/**Western Blot using Hamlet antibody was performed with rAAV2/1-minidysferlin injected and WT muscles and showed that the minidysferlin protein was correctly expressed in injected muscles. An equal amount of protein prepared from two minidysferlin-injected *Dysf ^prmd^ FDB* muscles previously shown to be able to reseal was added as control (left side of the blot). All parts of the image come from the same gel. A-actin was used as a loading control.
(TIF)
