# Supplementary figures and images for: Lack of Correlation between Outcomes of Membrane Repair Assay and Correction of Dystrophic Changes in Experimental Therapeutic Strategy in Dysferlinopathy
Source: PLoS One. 2012 May 29;7(5):e38036. doi: 10.1371/journal.pone.0038036 (PMC3362551; doi:10.1371/journal.pone.0038036)

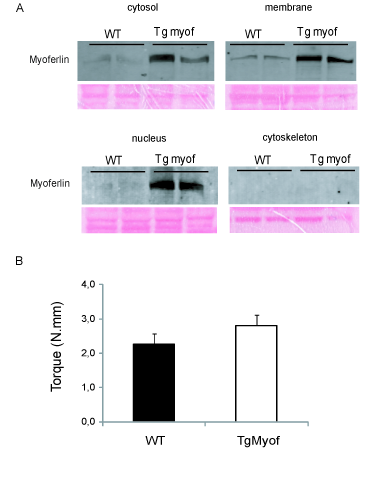

Supplement: Figure S1 — Characterization of TgMyof. A/Assessment of myoferlin levels in subcellular fractionations. The distribution of myoferlin in TA muscles was examined by Western analysis of subcellular fractions prepared using SPEK muscle extract from WT and TgMyof mice. Equal volumes of each fraction (nucleus, cytosol, membrane, cytoskeleton) were analyzed and show a similar distribution between fractions despite the overexpression (e.g. presence in the cytosol, nucleus and membrane fraction). B/Contractile torque of TgMyof and WT. The anterior compartment of the hindlimb of TgMyof and WT animals was tetanically stimulated for 300 ms and the contractile torques were measured. The differences were not statistically significant. (TIF) [file pone.0038036.s001.tif]

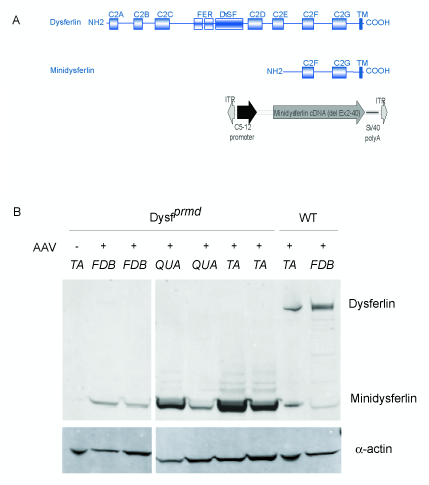

Supplement: Figure S2 — AAV vector for minidysferlin. A/Upper scheme: Scheme of the protein domain organization of the dysferlin protein with its 7 C2 (from C2A to C2G), Fer, Dysf and transmembrane domains. Middle scheme: Scheme of the minidysferlin used in this study. Lower scheme: Scheme of the rAAV construct for minidysferlin. B/Western Blot using Hamlet antibody was performed with rAAV2/1-minidysferlin injected and WT muscles and showed that the minidysferlin protein was correctly expressed in injected muscles. An equal amount of protein prepared from two minidysferlin-injected Dysf prmd FDB muscles previously shown to be able to reseal was added as control (left side of the blot). All parts of the image come from the same gel. A-actin was used as a loading control. (TIF) [file pone.0038036.s002.tif]
